# The clinical experience of the prognosis in opposite CEA and image change after therapy in stage IV colorectal cancer

**DOI:** 10.1038/s41598-022-24187-5

**Published:** 2022-11-22

**Authors:** Yi-Tien Su, Jia-Wan Chen, Shih-Ching Chang, Jeng-Kai Jiang, Sheng-Chieh Huang

**Affiliations:** 1 National Yang Ming Chiao Tung University, Taipei, Taiwan; 2 Far Eastern Memorial Hospital, No. 21, Sec. 2, Nanya W. Rd., Banqiao Dist., New Taipei City, 220 Taiwan; 3 Department of Mechanical Engineering, Asia Eastern University of Science and Technology, New Taipei City, Taiwan; 4 Division of Colon & Rectal Surgery, Department of Surgery, Taipei Veterans General Hospital, Taipei, Taiwan

**Keywords:** Cancer, Biomarkers, Gastroenterology, Medical research, Oncology

## Abstract

Carcinoembryonic antigen (CEA) levels and imaging are used to guide treatment for metastatic colorectal cancer (mCRC). This study evaluated changes in CEA and imaging findings in mCRC patients following systemic therapy and their clinical significance, especially the ones with inconsistent results of CEA and image findings. We enrolled 330 stage IV CRC patients who systemic therapy. Based on the Response Evaluation Criteria in Solid Tumors (RECIST) and a modification for CEA, patients were stratified into consistent and inconsistent response groups**.** Clinicopathological features and prognoses were compared between each groups. Different CEA/IMG groups showed no significant differences in terms of tumor location, initial CEA level, mucinous component, tumor differentiation and further surgical treatment rate. Inconsistent responses were observed in half of the patients (n = 165, 50%). The prognosis in the inconsistent groups with either CEA-SD or IMG-SD was dependent on the result of the other evaluation method (PR or PD). Cases with conflicting results between CEA and image groups (CEA-RD/IMG-PD, CEA-PD/IMG-PR) had an OS close to that of CEA-SD/IMG-SD (18.2 m, 16.2 m vs. 18.8 m, *P* = 0.620). The overall survival (OS) in the consistent (PR/PR ro PD/PD) groups were significantly different (*P* < 0.001). Combining CEA and imaging provides more information about mCRC patients who have undergone systemic therapy. Approximately half the patients have inconsistent responses, which is still valuable in predicting the prognosis.

## Introduction

Colorectal cancer (CRC) is a common disease around the world and is the third leading cause of cancer-related deaths in the United States and Taiwan^1,2^. However, in the recent years, new cancer prevention strategies and the development of medical technologies have resulted in a decrease in the incidence of CRC and disease mortality^3^.

Guidelines for treatment and follow-up of CRC patients have been established by different associations, including the National Comprehensive Cancer Network Guideline, American Society of Clinical Oncology Clinical Practice Guidelines, American Cancer Society Colorectal Cancer Survivorship Care Guidelines, and the European Society for Medical Oncology Clinical Practice Guidelines^4–7^. These guidelines include various recommendations for the surveillance and follow-up of CRC patients after treatment. Two important tools used to monitor the disease are imaging and tumor markers. The most applicable imaging technique is the CT scan, and the most widely used tumor marker is the carcinoembryonic antigen (CEA).

In cases of stage I to III CRC, the post-treatment follow-up is based on the complications from the previous therapy and early detection of recurrent tumors, and metachronous tumors. Although the different guidelines recommend different follow-up methods and intervals, there is no conflict between these methods. Some studies have shown better results by combining CEA analysis and CT scan for the follow-up of CRC^7–9^.

In stage IV, the follow-up and surveillance of CRC have different purposes. If curative resection of the CRC has not been achieved, its major goal is to monitor the treatment response and the possibility of tumor resection. The most popular modalities to monitor the treatment response recommended by the National Comprehensive Cancer Network, American Society of Clinical Oncology and European Society for Medical Oncology are CT scan and serum CEA^4,5,7^. With the use of combination therapy (5FU with Irinotecan or Oxaliplatin) and targeted therapies (e.g. Bevacizumab, Cetuximab, and Panitumumab), a significant improvement has been achieved in the response rates and survival in patients with advanced CRC^10–13^. Evaluating the treatment response thus becomes very important since there are many therapeutic combinations available to control metastatic diseases. The alterations in CEA levels and imaging findings in response to different treatments are usually consistent. However, there have been instances of inconsistencies that make further therapeutic decision making difficult.

In this study, we evaluated changes in imaging findings and serum CEA levels during follow-up of stage IV CRC patients who had undergone chemo/targeted therapy. For imaging studies, we used the Response Evaluation Criteria in Solid Tumors (RECIST) definition according to the World Health Organization (WHO) criteria to evaluate treatment response^14,15^. For serum CEA levels, we used a definition modified from the RECIST criteria to evaluate treatment response. Different findings from the imaging studies and CEA evaluation were correlated with overall survival (OS) to demonstrate different prognosis among each group.

## Methods

From 2010 to 2018, 719 stage IV CRC patients who received systemic therapy at the Taipei Veterans General Hospital were enrolled in this study. This study was approved by the Institutional Review Board of Taipei Veterans General Hospital (TPEVGH IRB No.: 2020-05-013AC) and was performed in accordance with the Declaration of Helsinki. The metastatic lesions were diagnosed using CT and/or MRI. If it became difficult to differentiate a metastatic lesion from a second primary tumor, a biopsy of the tumor was arranged to confirm the diagnosis. After the treatment is initiated, the follow up would be three months after the treatment including the physical examination, tumor marker and image. In order to compare the changes in CEA levels and imaging findings during systemic therapy, the findings before and after the sixth course of therapy were collected. Further surgical treatment with the primary lesion or metastatic lesion was arranged if the tumor was resectable following the systemic therapy, after discussing the findings at the multidisciplinary team combined conference. Patients without complete data or with CEA levels within the normal range (< 5 µg/L) at diagnosis were excluded from the study. In addition, some of the tumor lesions that were categorized as non-measurable according to the RECIST guidelines were also excluded. Finally, 330 patients were chosen for further evaluation.

Imaging for monitoring therapy response was performed using CT and/or MRI. Based on the findings, the response was stratified according to the RECIST guidelines as IMG-PR (partial response), IMG-SD (stable disease) and IMG-PD (progressive disease) (Table 1). CEA levels were checked at the Department of Nuclear Medicine at the Taipei Veterans General Hospital using a radioimmunoassay kit manufactured by CIS Biointernational at Gif-sur-Yvette in France. Based on the same grouping criteria of the RECIST guidelines, the changes in CEA levels following systemic therapy were stratified into three categories: CEA-RD (response disease), CEA-SD (stable disease), and CEA-PD (progressive disease). CEA levels decreased more than 30% of the original level indicated CEA response disease (CEA-RD); CEA increased more than 20% of the original level indicated CEA progressive disease (CEA-PD); the change of CEA does not fulfil the definition CEA-RD and CEA-PD were defined as CEA stable disease (CEA-SD) (Table 1).

**Table 1:** The definition of RECIST guideline for image and for CEA in this study.

RECIST guideline for image
Complete response (CR): Disappearance of all target lesions
Partial response (PR): Target lesions decrease > 30%
Stable disease (SD): Small change that do not fulfill PD and PD
Progressive disease (PD): Target lesions increase > 20% and sum of the increased target lesions > 5 mm or new lesion found

CEA change grouping according to RECIST guideline
CEA-RD (response disease): CEA decrease > 30%
CEA-SD (stable disease): CEA decrease ≤ 30% and increase ≤ 20%
CEA-PD (progressive disease): CEA increase > 20%

The study endpoint was OS between each evaluation groups of the patients. Based on the CEA and imaging findings after treatment, patients were categorized into nine groups: CEA-RD/IMG-PR, CEA-SD/IMG-SD, CEA-PD/IMG-PD (i.e. consistent response), CEA-RD/IMG-SD, CEA-RD/IMG-PD, CEA-SD/IMG-PR, CEA-SD/IMG-PD, CEA-PD/IMG-PR and CEA-PD/IMG-SD (i.e. inconsistent response). Clinicopathological features of the CEA-RD/IMG-PR, CEA-PD/IMG-PD (consistent response) and CEA-RD/IMG-PD, CEA-PD/IMG-PR (inconsistent response) groups were compared using the chi-square test. Numerical values were analyzed using the one-way ANOVA. Numbers were presented as a mean ± standard deviation. OS was presented as a survival curve and compared using the log-rank test. Statistical significance was defined as *P* < 0.05. Statistical analysis was performed using the Statistical Package and Service Solutions, IBM version 21.0.

### Ethics approval and consent to participate

This study was approved by the Institutional Review Board of Taipei Veterans General Hospital (TPEVGH IRB No.: 2020-05-013AC) and informed consent from each patient was waived.

## Results

A total of 330 patients were enrolled in this study, of which 199 (60.5%) were men and the median age at diagnosis was 62 years (range: 24–92 years). There were 81 (24.5%) right-sided colon cancers, 147 (44.5%) left-sided colon cancers and 102 (30.9%) rectal cancers (Table 2). Liver, lung and distant lymph node metastases were seen in 277 (83.9%), 63 (19.1%) and 14 (4.2%) patients, respectively. More than one site of distant metastasis was seen in 77 (23.3%) patients, and most of them (39, 50.6%) were combined liver and lung metastases. Seventy (21.2%) patients received further surgical treatment after chemo/target therapy and patients belonging to CEA-RD/IMG-PR, CEA-SD/IMG-PR and CEA-RD/IMG-SD groups had a higher probability to received further surgical treatment (27.6%, 30.7%, 26.7%, Table3).

**Table 2:** The relationship between treatment evaluation by image and CEA with different variables.

Variable	Study population	CEA-RD/IMG-PR n = 76	CEA-RD/IMG-PD n = 29	CEA-PD/IMG-PR n = 18	CEA-PD/IMG-PD n = 68	*P* value
Age [mean (years); range]	62 (24–92)	62 (31–94)	61 (28–88)	67 (51–91)	64 (73–89)	0.896
Male sex	199 (60.5%)	53 (69.7%)	16 (55.1%)	10 (55.5%)	48 (70.5%)	0.729
**Tumor location**						
Right side colon	81 (24.5%)	22 (28.9%)	7 (24.1%)	3 (16.7%)	18 (26.5%)	0.671
Left side colon	147 (44.5%)	31 (40.8)	13 (44.8%)	8 (44.4%)	30 (44.1%)	
Rectal cancer	102 (30.9)	23 (30.3%)	9 (31.1%)	7 (38.9%)	20 (29.4%)	
Initial CEA level (μg/L)	471.9 ± 1123.9	404.6 ± 1054.7	657.7 ± 1722.2	190.1 ± 173.9	355.2 ± 1006.3	0.736
Mucinous	22 (7.0%)	3 (3.9%)	2 (6.9%)	0 (0%)	6 (8.8%)	0.391
**Tumor differentiation**						
Well and moderate	276 (87.3%)	71 (93.4%)	25 (86.2%)	13 (72.2%)	55 (80.8%)	0.112
Poorly and undifferentiated	54 (12.7%)	5 (6.6%)	3 (10.3%)	4 (22.2%)	11 (16.2%)	
Further surgery	70 (21.1%)	20 (26.3%)	6 (20.7%)	2 (11.1%)	14 (20.6%)	0.778
Survival (months)	18.56 ± 2.1	27.9 ± 3.0	21.1 ± 2.8	18.1 ± 5.8	10.7 ± 0.9	< 0.01

**Table 3:** Further surgical treatment and the correlation with the treatment response by the image and the CEA.

Treatment response assess by image (n)	CEA-RD	CEA-SD	CEA-PD
CR	0	0	0
PR	21/76 (27.6%)	4/13 (30.7%)	1/18 (5.6%)
SD	17/60 (26.7%)	4/21 (19.0%)	4/30 (13.3%)
PD	6/29 (20.6%)	4/15 (26.6%)	9/68 (13.2%)

*CR* complete response, *PR* partial response, *SD* stable disease, *PD* progressive disease, *CEA-RD* response disease by CEA, *CEA-SD* stable disease by CEA, *CEA-PD* progressive disease by CEA.

In this study population, 24 (7.3%) patients received chemotherapy with 5FU + Leucovorin, 110 (33.3%) with FOLFOX, 83 (25.2%) with FOLFIRI, 18 (5.4%) with FOLFOX + Bevacizumab, 38 (11.5%) with FOLFIRI + Bevacizumab, 37 (11.2%) with FOLFOX + Cetuximab and 20 (6.1%) with FOLFIRI + Cetuximab. Patients who received chemotherapy combined with targeted therapy compared to those who received chemotherapy alone had more percentage in the CEA-RD/IMG-PR group (39.6% vs. 19.5%, Table 4) and less percentage in the CEA-PD/IMG-PD group (8.3% vs. 17.0%, Table 4) and showed a better disease prognosis (26.3 m vs. 15.8 m, *P* < 0.001).

**Table 4:** Combined target therapy or not and the correlation with the treatment response by the image and the CEA.

Treatment response assess by image (n)	Target + chemotherapy/chemotherapy		
	CEA-RD	CEA-SD	CEA-PD
CR	0	0	0
PR	39.6%/19.5%	8.3%/5.7%	6.3%/3.5%
SD	16.7%/18.8%	4.2%/7.8%	4.2%/8.5%
PD	10.4%/10.3%	2.1%/8.9%	8.3%/17.0%

*CR* complete response, *PR* partial response, *SD* stable disease, *PD* progressive disease, *CEA-RD* response disease by CEA, *CEA-SD* stable disease by CEA, *CEA-PD* progressive disease by CEA.

In reviewing the pathology of the primary tumors biopsy, 22 (7.0%) tumors were mucinous adenocarcinomas; five (1.5%) of them were well differentiated, 271 (82.1%) were moderately differentiated, 42 (12.7%) were poorly differentiated, and 12 (3.7%) were undifferentiated (Table 2). The median OS was 18.56 months, ranging from 2.3 to 135.2 months. Three hundred and seven patients (93.1%) died during the follow-up period. As shown in Table 5, the different groups based on the imaging and CEA findings showed significantly different survivals (IMG-PR: 25.5 m, IMG-SD: 22.1 m, IMG-PD: 12.3 m, *P* < 0.001; CEA-RD: 26.5 m, CEA-SD: 16.1 m, CEA-PD: 12.0 m, *P* < 0.001). CEA changes and imaging results showed a significant correlation using the Chi-square analysis (*P* < 0.001).

**Table 5:** Treatment response evaluated by the image and the CEA.

Treatment response assess by image (n)	Treatment response assess by CEA (n)				
	CEA-RD	CEA-SD	CEA-PD	Total	Survival (m)
CR	0	0	0	0	
PR	76	13	18	107	25.5
SD	60	21	30	111	22.1
PD	29	15	68	112	12.3
Total	165	49	116	330	
Survival (m)	26.5	16.1	12.0		*P* < 0.01

*CR* complete response, *PR* partial response, *SD* stable disease, *PD* progressive disease, *CEA-RD* response disease by CEA, *CEA-SD* stable disease by CEA, *CEA-PD* progressive disease by CEA.

**Figure 1:**
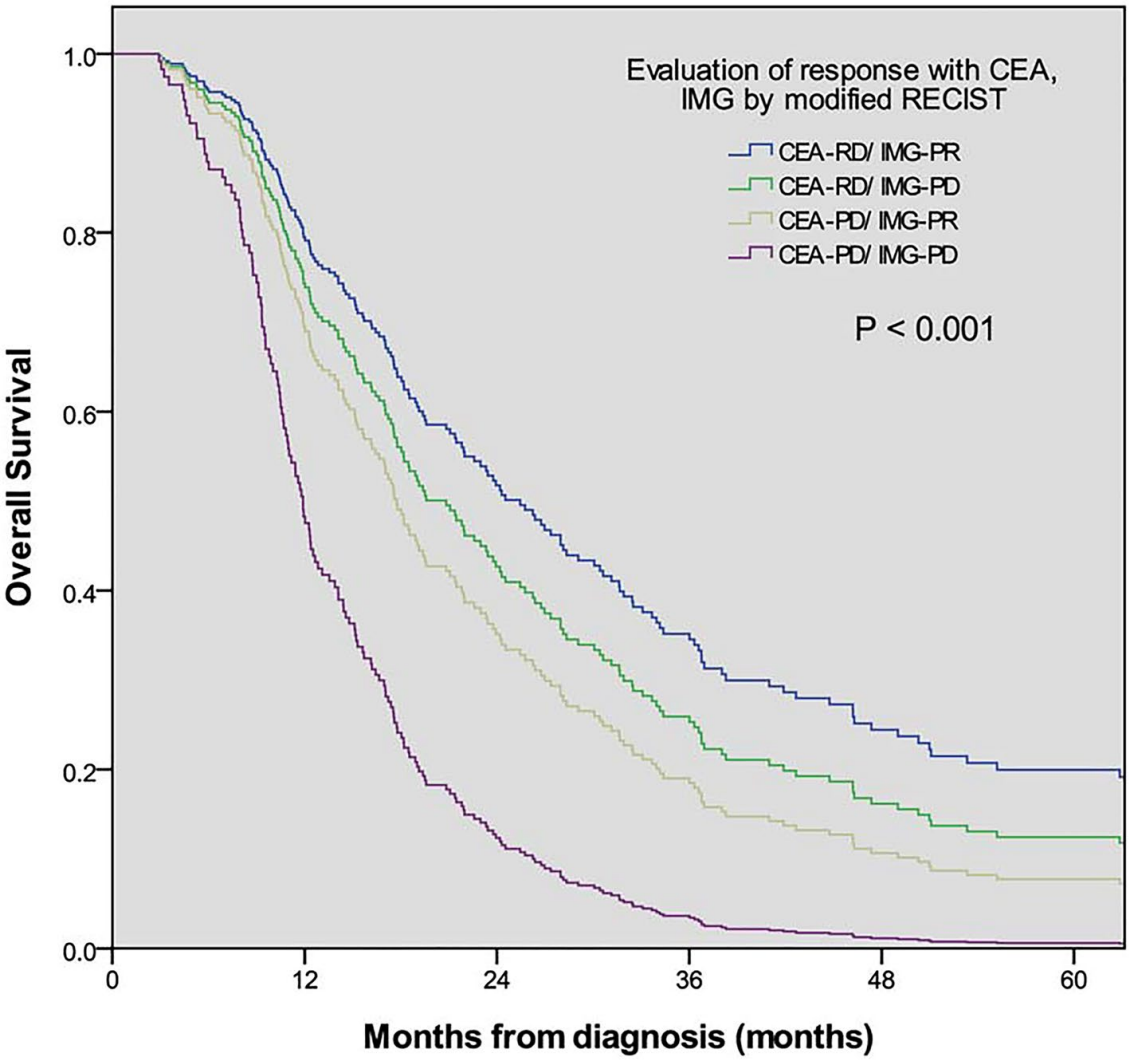
The survival curve between consistent and inconsistent results of CEA change and image findings. The OS in the CEA-RD/IMG-PR, CEA-RD/IMG-PD, CEA-PD/IMG-PR and CEA-PD/IMG-PD groups were 27.3 ± 6.6, 18.2 ± 4.8, 16.2 ± 3.8 and 10.7 ± 2.4 months, respectively and the differences were significant (*P* < 0.001).

The OS in the CEA-RD/IMG-PR, CEA-RD/IMG-PD, CEA-PD/IMG-PR and CEA-PD/IMG-PD groups were 27.3 ± 6.6, 18.2 ± 4.8, 16.2 ± 3.8 and 10.7 ± 2.4 months, respectively and the differences were significant (*P* < 0.001, Fig. 1). However, no significant differences were found between these groups in terms of primary tumor location, initial CEA level, mucinous component, tumor differentiation and further surgical treatment rate.

While 165 patients (50%) had consistent responses (CEA-RD/IMG-PR:76, CEA-SD/IMG-SD:21, CEA-PD/IMG-PD: 68), another 165 patients (50%) had inconsistent responses (CEA-SD/IMG-PR: 13, CEA-PD/IMG-PR: 18, CEA-RD/IMG-SD: 60, CEA-PD/IMG-SD: 30, CEA-RD/IMG-PD: 29, CEA-SD/IMG-PD: 15, Table 5). The OS in the consistent groups were 27.3 (CEA-RD/IMG-PR), 18.8 (CEA-SD/IMG-SD) and 10.7 (CEA-PD/IMG-PD) months with significantly difference (*P* < 0.001). The prognosis in the inconsistent groups with either CEA-SD or IMG-SD was dependent on the result of the other evaluation method. CEA-SD with IMG-PR had an OS of 24.2 months which was close to that of CEA-RD/IMG-PR (*P* = 0.618), while CEA-SD with IMG-PD had an OS of 11.5 months which was close to that of CEA-PD/IMG-PD (*P* = 0.701). Similarly, CEA-RD/IMG-SD had an OS of 26.4 months which was close to that of CEA-RD/IMG-PR (*P* = 0.851) and IMG-SD with CEA-PD had an OS of 15.3 months that was close to that of CEA-PD/IMG-PD (*P* = 0.064). Cases with conflicting results between CEA and image groups (CEA-RD/IMG-PD, CEA-PD/IMG-PR) had an OS close to that of CEA-SD/IMG-SD (18.2 m, 16.2 m vs. 18.8 m, *P* = 0.620).

## Discussion

This study is the one to compare different combinations of patient responses determined by CEA levels and tumor imaging in stage IV CRC patients following systemic therapy. Approximately half the patients had inconsistent responses indicating that more information should be added for the clinicians to evaluate the efficacy of treatment and adjust the regimen in a timely manner. We also observed a significant difference in the OS among the different CEA/IMG groups. It was not surprising to see the best survival (27.9 months) in the CEA-RD/IMG-PR group and the worst survival (10.4 months) in the CEA-PD/IMG-PD group. However, it was more interesting to find groups with inconsistent CEA and imaging findings. There were six groups with inconsistent responses in this study. Based on the survival data from these inconsistent response groups we divided them into two categories: groups combined with CEA-SD or IMG-SD, and groups with conflicting results between CEA analysis and imaging. The survival outcomes in the first category, where stable disease was determined by either imaging or CEA, was dependent on the results of the other detection method (CEA or imaging, respectively). If the other detection method showed a good response, the survival data was close to the consistent group of CEA-RD/IMG-PR, but if the results indicated disease progression, the survival data would be close to the consistent group of CEA-PD/IMG-PD. In the second category, wherein the CEA analysis and imaging had conflicting findings (CEA-RD/IMG-PD, CEA-PD/IMG-PR), the survival data were close to the CEA-SD/IMG-SD group. This is an interesting finding that demonstrates the relationship between changes in CEA levels and imaging results and their importance in the evaluation of response to systemic therapy in CRC. The results, especially the survival outcomes for groups with inconsistent CEA and imaging findings could aid clinicians in predicting the prognosis of stage IV patients receiving therapy. Several clinicopathological traits were included for analysis, and none of them showed significant correlation with the CEA and imaging findings following systemic therapy.

Tumor evaluation provides information that helps the clinician judge the disease status and prognosis and decide on a treatment strategy. In stage IV CRC, treatment is mainly based on systemic therapy and tumor resection, including primary tumor resection and metastasectomy. Evaluating the response to a systemic treatment is important in stage IV CRC because the prognosis is highly correlated with it and the clinician would consider the response and the adverse effects together. There are many ways to evaluate a treatment response, which range from a clinical physical examination and colonscopy assessment to a tumor marker follow-up and radiological imaging studies and nuclear positron emission tomography (PET) survey^16^. In order to evaluate and grade the responses, RECIST was proposed in 2000 and modified in 2009^15^. RECIST standardized the treatment responses determined by imaging, for the clinician to judge and compare them and predict the prognosis. According to the RECIST guidelines, other methods of evaluation such as tumor markers can also aid in the accurate evaluation of treatment response, especially for detection of new metastatic or recurrent lesions. However, there are no guidelines for grading changes in tumor markers following therapy. As per the RECIST guidelines, we defined three categories of the disease based on changes in the levels of tumor markers following therapy: response disease RD, stable disease SD, and progressive disease PD. Changes in CEA levels not exceeding 20% of the original levels indicated stable disease. According to this categorization, the OS for CEA-RD, CEA-SD and CEA-PD were 26.5 m, 16.1 m, and 12 m, respectively with significant differences (*P* < 0.001).

CEA is a glycosylated cell surface glycoprotein that was first reported by Gold and Freedman in 1965^17^. Initially, thought to be a tumor marker for CRC, it is now found to be related to a variety of benign and malignant diseases^18^. The role of CEA in CRC has been widely studied. Mayer et al. were the first to show the relationship between CEA levels and response to chemotherapy in CRC^19^, which has since then been confirmed by several studies^20–24^. Decrease in CEA levels following adjuvant therapy in CRC leads to a better response to treatment and better prognosis. On the other hand, increase in CEA levels following the treatment predicts poor response to therapy and therefore a poor prognosis. However, there are still some limitations to using CEA as a tumor marker for follow-up of CRC. Previous studies have shown that not all CRC patients have elevated CEA levels^25^. In stage IV CRC, around 30% of the patients do not have elevated CEA and could not be used as a follow-up marker after therapy^25,26^. Besides, some physiological conditions such as impaired liver function especially during and after a hepatotoxic chemotherapy could affect the CEA levels in CRC patients. Heavy smokers also show changes in serum CEA levels regardless of having cancer^27^. CEA could act as a marker for the follow-up of CRC, but none of the guidelines suggest using it alone. Alternatively, combining it with other tools such as imaging could provide more information to evaluate the response to therapy.

Consistent with earlier reports, we also found a correlation between response to therapy and CEA levels and imaging results in CRC (*P* < 0.001)^14,28,29^. These two detection methods, therefore play an important role in the follow-up of CRC. However, discrepancies between these two methods is not a rare situation, and in our study population, half of the patients had inconsistent findings using the two methods. Fakih, M. G., and Padmanabhan, A. have reported a decrease in CEA levels with progression of a tumor in a CT scan^30^. They attributed this to tumor dedifferentiation or the selective result of non-CEA-producing tumors following chemotherapy. Besides, tumor lysis and changes in its morphology following therapy would also affect the interpretation of the actual tumor condition from the images. On the other hand, increase in CEA levels with tumor regression in imaging findings can be explained by the limitation of the imaging in detecting disseminated metastases, or small recurrent tumors that could not be easily identified. Both these discrepant situations indicate that each of the methods has its limitations in evaluating the treatment response, and their combined use could decrease the risk of false positives and negative findings. The causes that contribute to the discrepancy between CEA measurements and imaging need to be further studied.

The limitation of this study is that it is a retrospective survey. Some groups such as CEA-SD/IMG-PR had small patient numbers. More data collection for these groups would strengthen the survival results. Patients who could not be evaluated for CEA or by imaging were excluded from the study. Other methods to evaluate treatment response are needed for such patients.

## Conclusion

Follow-up of mCRC after chemotherapy is important to evaluate the response to therapy and disease status. CEA measurement and imaging are two most popular follow-up methods in clinical practice. In our study, around half of the CRC patients receiving chemotherapy did not have the same results by measuring CEA levels and imaging.

According to the survival data, patients showing a good response to treatment by both CEA levels and imaging had the best survival, whereas those who showed a poor response by both these methods had the worst survival. CEA-SD/IMG-SD group had an OS that was intermediate of the survivals for CEA-RD/IMG-PR and CEA-PD/IMG-PD groups. The survival outcomes in discrepant groups where stable disease is suggested by one evaluation method are related to the results of the other method. The outcomes in cases with conflicting results between CEA measurements and imaging are close to that of the CEA-SD/IMG-SD group. This study provides prognostic information based on different results from CEA measurements and imaging studies in CRC, thereby helping the clinician predict the status of the therapy by CEA and imaging.

## Data Availability

The data of this study are available from the first author and corresponding author upon reasonable request.

## Abbreviations

CEA: Carcinoembryonic antigen
mCRC: Metastatic colorectal cancer
RECIST: Response evaluation criteria in solid tumors
OS: Overall survival
CRC: Colorectal cancer
WHO: World health organization
IMG-PR: Image partial response
IMG-SD: Image stable disease
IMG-PD: Image progressive disease
CEA-RD: CEA response disease
CEA-SD: CEA stable disease
CEA-PD: CEA progressive disease
PET: Positron emission tomography
